# TPepPro: a deep learning model for predicting peptide–protein interactions

**DOI:** 10.1093/bioinformatics/btae708

**Published:** 2024-11-25

**Authors:** Xiaohong Jin, Zimeng Chen, Dan Yu, Qianhui Jiang, Zhuobin Chen, Bin Yan, Jing Qin, Yong Liu, Junwen Wang

**Affiliations:** School of Electronic Information, Guangxi University for Nationalities, Nanning 530000, China; Division of Applied Oral Sciences and Community Dental Care, Faculty of Dentistry, The University of Hong Kong, Hong Kong SAR, China; Division of Applied Oral Sciences and Community Dental Care, Faculty of Dentistry, The University of Hong Kong, Hong Kong SAR, China; Division of Applied Oral Sciences and Community Dental Care, Faculty of Dentistry, The University of Hong Kong, Hong Kong SAR, China; School of Pharmaceutical Sciences (Shenzhen), Shenzhen Campus of Sun Yat-sen University, Shenzhen, Guangdong 518107, China; Division of Applied Oral Sciences and Community Dental Care, Faculty of Dentistry, The University of Hong Kong, Hong Kong SAR, China; School of Pharmaceutical Sciences (Shenzhen), Shenzhen Campus of Sun Yat-sen University, Shenzhen, Guangdong 518107, China; School of Artificial Intelligence, Guangxi University for Nationalities, Nanning 530000, China; Division of Applied Oral Sciences and Community Dental Care, Faculty of Dentistry, The University of Hong Kong, Hong Kong SAR, China; State Key Laboratory of Pharmaceutical Biotechnology, The University of Hong Kong, Hong Kong SAR, China; HKU Shenzhen Hospital, Shenzhen 518000, China

## Abstract

**Motivation:**

Peptides and their derivatives hold potential as therapeutic agents. The rising interest in developing peptide drugs is evidenced by increasing approval rates by the FDA of USA. To identify the most potential peptides, study on peptide-protein interactions (PepPIs) presents a very important approach but poses considerable technical challenges. In experimental aspects, the transient nature of PepPIs and the high flexibility of peptides contribute to elevated costs and inefficiency. Traditional docking and molecular dynamics simulation methods require substantial computational resources, and the predictive accuracy of their results remain unsatisfactory.

**Results:**

To address this gap, we proposed TPepPro, a Transformer-based model for PepPI prediction. We trained TPepPro on a dataset of 19,187 pairs of peptide-protein complexes with both sequential and structural features. TPepPro utilizes a strategy that combines local protein sequence feature extraction with global protein structure feature extraction. Moreover, TPepPro optimizes the architecture of structural featuring neural network in BN-ReLU arrangement, which notably reduced the amount of computing resources required for PepPIs prediction. According to comparison analysis, the accuracy reached 0.855 in TPepPro, achieving an 8.1% improvement compared to the second-best model TAGPPI. TPepPro achieved an AUC of 0.922, surpassing the second-best model TAGPPI with 0.844. Moreover, the newly developed TPepPro identify certain PepPIs that can be validated according to previous experimental evidence, thus indicating the efficiency of TPepPro to detect high potential PepPIs that would be helpful for amino acid drug applications.

**Availability and implementation:**

The source code of TPepPro is available at https://github.com/wanglabhku/TPepPro.

## Graphical abstract

**Graphical abstract:**
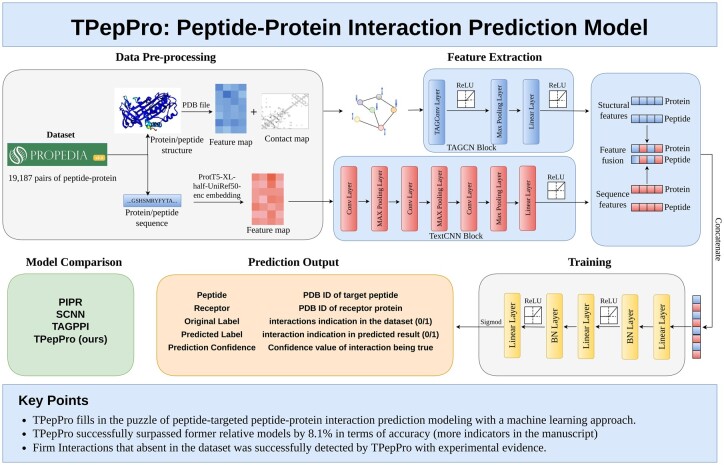

## 1 Introduction

Peptide-protein interactions (PepPIs) refer to interactions between proteins and peptide molecules that are ubiquitous in living organisms and involved in many biological processes. The specificity and biological activity of peptides make them a good starting point for new treatments. Identifying accurate PepPIs is critical to the invention of such treatments, but determining PepPIs experimentally is often time-consuming and expensive. Predicting whether they have interactions is of great significance for the development of peptide drugs. To address this problem, numerous computational methods have been developed to predict the relationship between proteins and peptides (Cunningham *et al.* 2020).

Recently, rapidly developing deep learning techniques have provided viable solutions for modelling protein-ligands or protein–protein interactions (PPIs) with better accuracy while requiring fewer computational resources (Liu *et al.* 2018, Tang *et al.* 2023, Yang *et al.* 2023a). However, the advancement in machine learning has not yet significantly impacted PepPI research. To date, *in silico* research targeting peptides has primarily focused on peptide-protein docking or molecular dynamic simulations (Keeble *et al.* 2019, Lee *et al.* 2019, Johansson-Åkhe *et al.* 2020, Sunny and Jayaraj 2022). Predictions based on conventional docking have been reported to fail biologically activity tests (Cole *et al.* 2005, Ramírez and Caballero 2016). Previous studies have utilized machine learning methods that widely applied in vision tasks to construct PepPI models, such as Convolutional Neural Network (CNN) (Ballester and Mitchell 2010, Yin *et al.* 2023). However, in terms of PepPIs, features include not only structural but also sequential.

Fortunately, artificial intelligence models have experienced rapidly updated in recent years (Sinha *et al.* 2023, Yang *et al.* 2023b, Li *et al.* 2024). The advantage of Transformer in amino acid sequence analysis is its ability to capture long-distance dependencies and to effectively handle long sequence data. Meanwhile, through the self-attention mechanism, it can automatically learn the important features in the sequences and improve the prediction performance. Here, we employ a transformer-based model with enhanced capability to comprehend contextual information in protein sequences and to construct more accurate and reliable PepPI prediction models. Moreover, in our study, we paid special attention to whether the prediction results could be experimentally validated. Despite the need for further experimental confirmation, we still find experimental evidence beyond the scope of the training and testing sets to confirm the effectiveness of the TPepPro model in practical applications.

Although there are growing interests in making peptide drugs and increasing number of approved peptide therapies, only a handful of work has been proposed to utilize machine learning or deep learning-based methods to model PepPIs. Hence, there is a pressing need for more advanced machine learning or deep learning-based models with superior efficiency for discovering PepPIs, specifically tailored for predicting PepPIs. In this study, we propose a novel model named TPepPro, that combines features extracted from both local protein sequences and global protein structures. The TPepPro system optimizes the architecture of structural featuring neural network with BN-ReLU (Batch Normalization—Rectified Linear Unit). We applied a 5-fold cross-validation to evaluate the performance of TPepPro as compared with other models, including PIPR (Chen *et al.* 2019), SCNN (Wang *et al.* 2019), and TAGPPI (Song *et al.* 2022). Our findings show an enhanced prediction accuracy of 0.855, a notable improvement over TAGPPI, the second-best model which achieved 0.774, and an increase of 8.1%. More importantly, the TPepPro method can identify PepPIs, consistent with those validated in previous experiments. Therefore, these findings demonstrate the superior ability of our method to discover potential PepPIs that would be helpful for amino acid drug applications.

## 2 Materials and methods

### 2.1 Workflow of TPepPro

**Figure 1.**
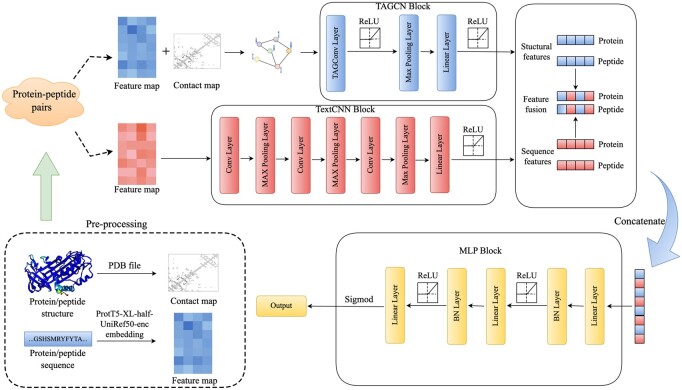
Architecture of TPepPro. (1) Data preprocessing: The amino acid sequences corresponding to proteins and peptides are encoded as vectors of size *L* × 1024. Here, *L* represents the length of the protein/peptide amino acid sequence, and 1024 denotes that each amino acid is encoded into a vector of 1024 dimensions. Additionally, distances between residues in the PDB-format protein and peptide structure files are calculated to obtain the contact map files of proteins and peptides. (2) TextCNN module is utilized to extract local sequence features of proteins and peptides. (3) Extraction of protein and peptide structural features: The TAGCN module is used to extract structural features through the contact map files of proteins and peptides. (4) Prediction module: Finally, the local sequence features and structural features of the given protein-peptide pairs obtained from the above steps are fused before being input into the prediction module. The generated prediction results undergo a sigmoid activation function for nonlinear transformation, resulting in an output between 0 and 1. Based on a set threshold, here set as 0.5, the presence of interaction in the input peptide-protein pairs is determined. Pairs with output results greater than or equal to 0.5 are considered to have interaction; pairs with output results less than 0.5 are deemed to have no interaction.

The TPepPro model proposed in this study uses an end-to-end deep learning method. There are four modules in TPepPro (Fig. 1): (i) Data pre-processing: Protein sequences are processed by ProtTrans, which is based on the Transformer architecture. This model has proven to be an excellent model on encoding the syntax and semantics of protein sequences (Elnaggar et al. 2022). The highest performance model of ProtTrans, ProtT5_XL_half_UniRef50-enc, is utilized in this pipeline. Structural features are encoded as contact map. (ii) Extracting local features of protein sequences using TextCNN (Kim 2014). (iii) Extracting protein structural features using TAGCN (Du *et al.* 2018). Compared with the original GCN, TAGCN is chosen for its better performance and accuracy as it uses a set of filters that are specific for each node. (iv) Prediction Model. In the training module, the Batch Normalization layer was placed prior to the ReLU layer (Ioffe and Szegedy 2015). This is because the BN layer can make the mean of the input values to be 0 and the variance to be 1, alleviating the problem of vanishing gradient of the ReLU function to a certain extent. This setting helps to keep the gradient passing efficiently while training the neural network, and to improve the performance (Garbin *et al.* 2020).

### 2.2 Data collection

Five datasets are utilized in this study (Supplementary Table S1), comprising: (i) The protein-peptide complex dataset Propedia v2.3 (Martins *et al.* 2023); (2) The human protein dataset DIP (Zhao *et al.* 2022); (3) The yeast protein dataset (Salwinski *et al.* 2004); (4) The neocoronavirus-human protein dataset (Yang *et al.* 2021). (v) The HIV-human protein dataset (Yang *et al.* 2021). The protein-peptide complex dataset from Propedia v2.3 serves as the primary dataset in this research.

### 2.3 Data pre-processing

Developed by DeepMind Google, ProtTrans uses the self-attention mechanism optimized for understanding the syntax and semantics of protein sequences (Elnaggar *et al.* 2022). ProtT5-XL-UniRef50 is chosen here for representing proteins with vectors as it was the best performance among ProtTrans models. To speed this step up, the model’s half-precision mode is turned on, namely, ProtT5-XL-half-UniRef50-enc. It was verified by Elnaggar *et al.* that this modification does not compromise the performance.

**Figure 2.**
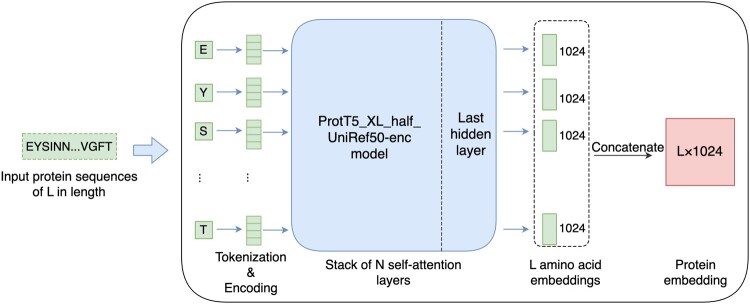
Protein amino acid embedding generation diagram. Protein sequences are first subjected to tokenization and positional encoding, followed by generating context-aware embeddings for each amino acid using the ProtT5-XL-half-UniRef50-enc model, resulting in vector representations of the proteins.

As shown in Fig. 2, we firstly tokenize and encode the input protein sequences. The encoded vectors are piped into ProtT5-XL-half-UniRef50-enc model, creating context-aware embeddings for each token. The vector representation of the protein is therefore obtained with the output being $X\in R^{L\times 1024}$. *L* represents the number of amino acids of the input protein.

### 2.4 Strategy of extracting local feature of protein sequence

This research adopts a TextCNN module for extracting local sequence features of proteins (Zhao *et al.* 2022). The module consists of three CNN layers and three max pooling layers. This structure is designed to achieve effective feature extraction and classification of protein sequences through the combination of CNNs and max pooling layers, while ensuring computational efficiency and robustness at the same time. Detailed extraction process is described below:

Firstly, ProtT5-XL-half-UniRef50-enc model is used to encode protein sequences into vector representations, denoted as $X\in R^{L\times 1024}$. Next, to ensure a fixed output vector size for the TextCNN module, the maximum length of the protein sequence *L* is set to be 1200. When a protein sequence is less than 1200 in length, a zero-padding approach would be applied to complement the sequence to fit 1200. Therefore, the formatted vector as $X\in R^{1200\times 1024}$ is adopted as further input of TextCNN.

The first convolutional layer of TextCNN has 128 output channels and a convolutional kernel size of 3. The output feature map has a size of 1198 × 128. This feature map was then fed into a maximum pooling layer with a step size of 3, resulting in another feature map of the protein with a size of 399 × 128. The processes mentioned above are repeated twice. Eventually, local feature vectors of size $F_{s1}\in R^{1\times 128}$ and $F_{s2}\in R^{1\times 128}$ will be output from TextCNN module as the protein sequence features.

### 2.5 Extracting protein structure features

Protein structural features are extracted through the construction of a protein’s contact graph. First, a contact graph file of the protein is constructed based on its structure file (PDB file). Bio.PDB, a module in the BioPython package, is used to process protein structure files in PDB format and calculate distances between residues (Hamelryck and Manderick 2003). The contact graph is square shaped with dimensions *L* × *L*. From this contact graph, we derive the Adjacency Matrix *A* and the Node Feature Matrix *X*. The adjacency matrix, denoted as $A\in R^{L\;\times \;L}$, represents connections between nodes. A value of 0 indicates no connection between two nodes, while a value of 1 indicates the presence of a connection. The node feature matrix, represented as $X\in R^{L\times 1024}$, consists of feature vectors of 1024 dimensions for each node.

Subsequently, the obtained adjacency matrix *A* and the node feature matrix *X* are input into the TAGCN layer, a variant of GCN. Traditional GCN sets *K* = 1 after approximating filters with Chebyshev polynomials, while TAGCN employs *K* filters to extract local features of varying sizes, with *K* serving as a hyperparameter. *K* varies among filters, ranging from 1 to *K*, akin to GoogleNet’s filters. The convolution process of TAGCN is demonstrated as follows:

Here, the graph convolution on the first hidden layer is demonstrated as an example, with the resulting pattern applicable to any other hidden layers. In this demonstration, it is assumed that $C_{l}$ features are mapped to each node. Subsequently, the adjacency matrix underwent self-looping and normalization:

(1) $$A=D^{{-}_{2}^{1}}\bar{A}D^{{-}_{2}^{1}}.$$

$G_{c,f}^{\left(l\right)}\in R^{N_{l}\times N_{l}}$ denote the form of *f*th graph filter. The convolution of a graph is the product of a matrix and a vector, namely, $G_{c,f}^{\left(l\right)}x_{c}^{\left(l\right)}$. Therefore, the output feature map after the *f*th map filter is:

(2) $${\;y}_{f}^{\left(l\right)}=\sum_{c=1}^{C_{l}}G_{c,f}^{\left(l\right)}x_{c}^{\left(l\right)}+b_{f}^{\left(l\right)}1_{N_{l}}.$$

In form (2): $G_{c,f}^{\left(l\right)}=\sum_{k=0}^{K}g_{c,\;f,k}^{\left(l\right)}A^{k}$. $g_{c,f,k}^{\left(l\right)}$ represents the graph filter polynomial coefficients. $b_{f}^{\left(l\right)}$ is the learnable bias. $1_{N_{l}}$ means all elements of the *N*-dimensional vector are 1.

According to the CNN architecture, an additional nonlinear operation would be employed after the convolution operation for each graph.

(3) $$x_{f}^{\left(l+1\right)}=\sigma \left(y_{f}^{\left(l\right)}\right).$$

Afterwards, the protein structural features extracted by TAGCN are fed into the maximum pooling layer and the linear layer containing 128 neurons. This process ensures a fixed number of outputs from the feature extraction module. Finally, for a pair of protein spatial maps $G_{i}$ and$\;G_{j}$, we extract its structural feature vectors as $F_{g1}\in R^{1\times 128}$ and$\;F_{g2}\in R^{1\times 128}$, respectively.

**Figure 3.**
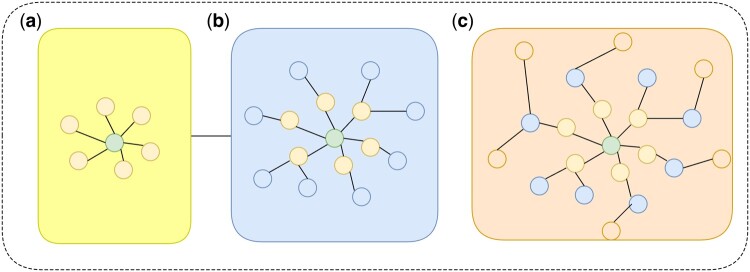
Topology-Adaptive Graph Convolutional Network (TAGCN) convolution process with K=3.The central amino acid node aggregates features from neighboring nodes within three hop distances (K=1,2,3), enabling the capture of both local and extended structural information.

The convolution process of TAGCN at *K* = 3 (Fig. 3) is illustrated below. The feature map of each vertex is assumed to contain one feature. Similarly, CNN, multiple channels are formed from the features extracted by multiple filters in each convolutional layer. Features extracted in filter ranging from 1 to 3 are denoted as$\;g_{c,f,1}^{\left(l\right)}A^{1}x^{\left(l\right)}$, $g_{c,f,2}^{\left(l\right)}A^{2}x^{\left(l\right)}$ and $g_{c,f,3}^{\left(l\right)}A^{3}x^{\left(l\right)}$. Features extracted by the three filters represent the relationship between the vertices and their neighbors in different spatial ranges. The new features of the green vertices in the graph are obtained by linearly combining them. New features of the vertex are denoted as $y_{f}^{\left(l\right)}=g_{c,f,1}^{\left(l\right)}A^{1}x^{\left(l\right)}+g_{c,f,2}^{\left(l\right)}A^{2}x^{\left(l\right)}+g_{c,f,3}^{\left(l\right)}A^{3}x^{\left(l\right)}+b_{f}^{\left(l\right)}1_{N_{l}}$.

### 2.6 Prediction module

The local sequence features ($F_{s1}$ and $F_{s2}$) and structural features ($F_{g1}$ and $F_{g2}$) of the proteins obtained from the above steps are fused in the prediction module, based on the formulas:

(4) $$F_{gc1}=\left(1-w\right)F_{g1}+wF_{s1}.$$

(5) $$F_{gc2}=\left(1-w\right)F_{g2}+wF_{s2}.$$

The fused features are connected and fed into the MLP layer, which is stacked of three linear layers (FC layer). The first two FC layers are followed by the BN function and ReLU activation function. Whereas the prediction results generated by the last FC layer are nonlinearly transformed by the Sigmoid activation function, obtaining an output that lies between 0 and 1. A judgment of whether the input protein pair interacts or not is then obtained based on the set threshold. Here the threshold is set to 0.5. Namely, an output prediction scores greater than or equal to 0.5 indicates that the input protein pairs are interacting, and vice versa.

### 2.7 Evaluation metrics

Five-fold cross-validation is used to evaluate binary prediction from seven criteria, namely accuracy (ACC), precision (Prec), recall (Recall), specificity (Spec), F1-score (F1), Area Under Curve (AUC), and the area under PRC curve (The area under the precision-recall curve, AUPRC).

(6) $$\mathrm{ACC}=\frac{\text{TP}+\text{TN}}{\text{TP}+\text{FN}+\text{FP}+\text{TN}},$$

(7) $$\text{Prec}=\frac{TP}{\text{TP}+\text{FP}},$$

(8) $$\text{Recall}=\frac{\text{TP}}{\text{TP}+\text{FN}},$$

(9) $$\mathrm{Spec}=\frac{\text{TN}}{\text{TN}+\text{FP}},$$

(10) $$F1=\frac{2*\text{Prec}*\text{Recall}}{\text{Prec}+\text{Recall}}.$$

In the above formula: TP (True Positive) represents that the prediction is positive, and the prediction is correct; TN (True Negative) means that the prediction is negative, and the prediction is correct. FP (False Positive) means that the prediction is positive, but the prediction is incorrect; FN (False Negative) indicates that the prediction is negative, but the prediction is incorrect.

(11) $$\mathrm{FPR}=\frac{\mathrm{FP}}{\mathrm{FP}+\mathrm{TN}},$$

(12) $$\mathrm{TPR}=\frac{\mathrm{TP}}{\mathrm{TP}+\mathrm{FN}}.$$

AUC is the area enclosed by the ROC curve and the *x*-axis, the ROC curve *x*-axis is FPR (False Positive Rate), and the *y* axis is TPR (True Positive Rate).

AUPRC is the area enclosed by the PRC curve and the *x*-axis, the PRC curve *x*-axis is the recall (Recall), and the *y*-axis is the precision (Prec).

### 2.8 Interpretability analysis

The interpretable analysis of the significant global features of protein structure extracted by TAGCN involves the following process: First, we load the amino acid sequences of the proteins, along with their contact maps and encoded vector representations. Next, we generate an adjacency matrix based on the contact map and create a graph data structure, incorporating the protein-encoded vectors as node features. These data are then input into the TAGCN model. The input graph data undergoes processing through the TAGConv layers, where graph convolution operations update the node features, followed by a forward propagation to obtain the output features processed by the TAGCN model. Finally, these output features are passed through an attention mechanism module to derive the corresponding attention scores, which are then combined to calculate the node importance scores.

To interpret analysis of the crucial local features of protein sequences extracted by TextCNN, we utilized visualizations of the activation maps of the convolutional kernels. These maps provide effective means to understand how TextCNN captures features from the input data. In the context of TextCNN, the activation maps illustrate the activation values generated as the convolutional kernels slide over the protein sequences. These activation values reflect the degree of matching between the kernels and the local features at different positions within the input sequence.

### 2.9 Model training

TPepPro takes the protein/peptide sequence features and predicts contact maps as input. We used the dgl libraries of Python 3.7, PyTorch 1.5.1, and CUDA 10.1 to implement TAGCN (Du *et al.* 2018). At the same time, the experiment took advantage of the powerful computing of the GPU, namely NVIDIA Quadro RTX, 24GB memory. The TPepPro model was trained on 50 epochs on the protein-peptide complex dataset using Adam optimizer, with a learning rate and batch size of 0.001 and 32, respectively. To avoid overfitting, BN technology is used during training. Other parameters took the default values provided by PyTorch (Zhai *et al.* 2023).

## 3 Results

The TPepPro model is composed of an investigation into the effects of sequence encoding methods, graph CNN methods, and the architecture of the neural network. Comparative analyses are conducted between the TPepPro model and other state-of-the-art methods. The performance of the TPepPro model on different datasets is examined using ROC curves and PR curves. Finally, case studies of high-confidence results are provided with experimental evidence.

### 3.1 Dataset

The protein-peptide complex dataset is from Propedia database (http://bioinfo.dcc.ufmg.br/propedia2/index.php/download). The latest version v2.3 contains 49 300 protein-peptide complexes. We treated protein-peptide complexes as positive protein-peptide samples, and negative interactions are constructed by randomly pairing protein-peptide pairs and not present in positive dataset. There may be inconsistencies between the number of amino acids in the contact diagram generated from their corresponding structure files and the number of amino acids in the embedding generated from their original amino acid sequences. This discrepancy arises from variations in the representation of the protein structure and the raw amino acid sequence, potentially impacting the analysis and interpretation of the data. At this time, the amino acid embedding of the protein cannot correspond to the amino acids in the contact map, and the amino acid vertices in the diagram generated by the contact diagram cannot obtain the corresponding amino acid expression, so that the subsequent extraction of protein structure features cannot be carried out. Therefore, we only selected proteins and peptides whose amino acid numbers in the contact chart matched their original amino acid numbers. As shown in Table 1, there were 9594 pairs of positive peptide-protein complex samples and 9593 pairs of negative samples, including 14 374 types of valid proteins and 9594 valid peptides.

**Table 1.** Dataset statistics.

Name	Total number of samples	Types of protein	Types of peptide	Positive samples	Negative samples
Protein-peptide dataset	19 187	14 374	9594	9594	9593

### 3.2 Evaluation on sequence coding strategy

**Figure 4.**
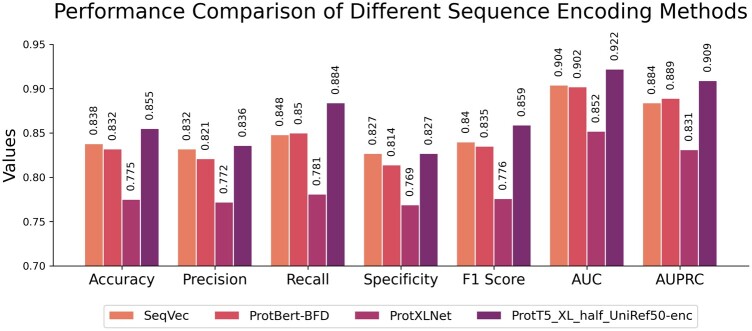
Evaluation of different sequence coding methods. The horizontal axis represents statistical parameters of model accuracy. Colors represent different models. The vertical axis values represent scores obtained by different models for evaluation.

To verify the robustness of the sequence coding method used in this study, a set of comparative tests was designed. The comparison of the ProtT5_XL_half_UniRef50-enc coding method with SeqVec (Heinzinger *et al.* 2019), ProtXLNet, and ProtBert-BFD (Elnaggar *et al.* 2022). To ensure the reliability of this experiment, the sequence coding strategy was controlled as the sole variable. The model architecture used in this experiment is TPepPro. Our data experiments compared the dataset consists of protein-peptide complex that sourced from Propedia v2.3. As shown in Fig. 4, model ProtT5_XL_half_UniRef50-enc we used here ranked the first in all evaluate parameter. The robustness of ProtT5_XL_half_UniRef50-enc coding method has been justified.

### 3.3 Evaluation on structural featuring strategy

To showcase the advantages of the graph CNN employed in this study for extracting protein structural features, we conducted a series of comparative experiments. The other graph CNNs used for this purpose include GAT (Veličković *et al.* 2018), APPNP (Gasteiger *et al.* 2022), and GATV2 (Brody *et al.* 2022), alongside the TAGCN (Du *et al.* 2018) utilized in this research (where the graph CNN for protein structural feature extraction is a single layer).

**Figure 5.**
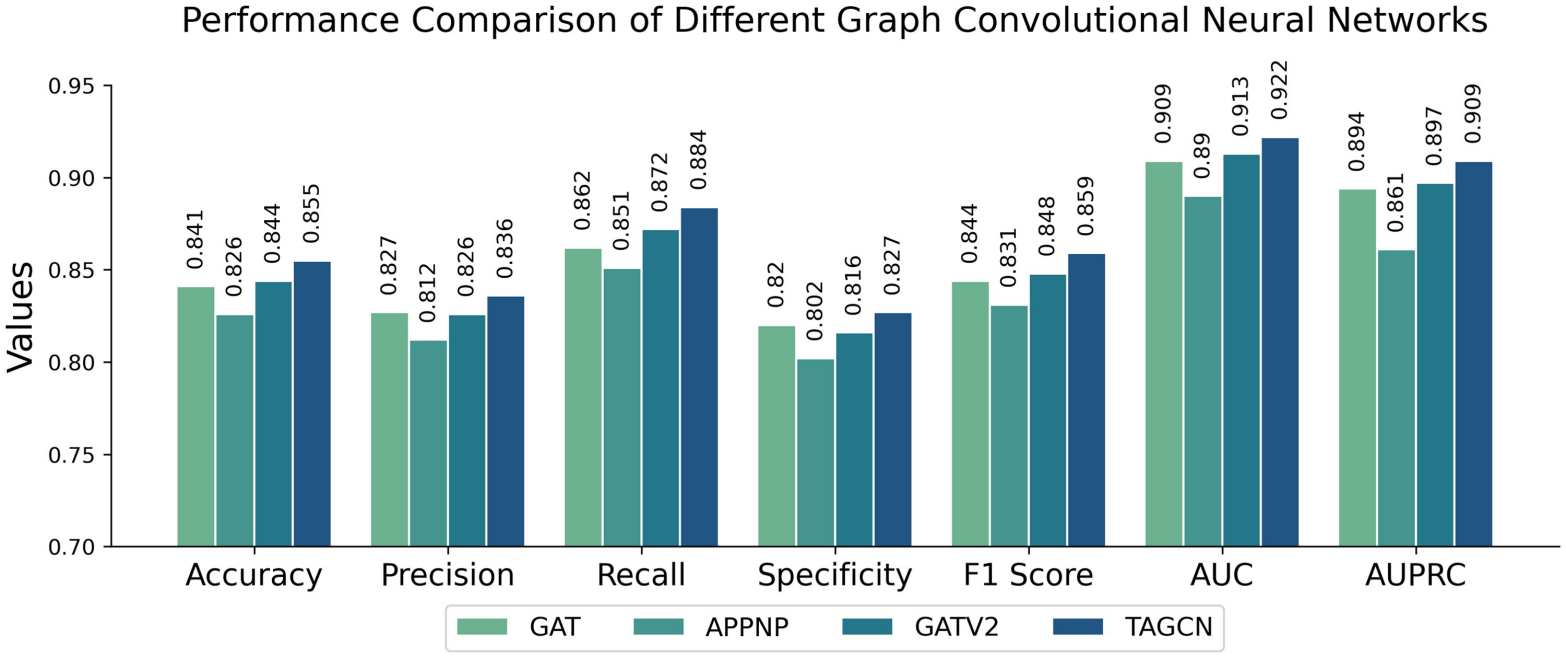
Evaluation of different methods for extracting protein structural features. The figure legend is the same as Fig. 4.

Utilizing the same dataset (protein-peptide complex), we employed TPepPro for predictive modeling and facilitated direct comparative analysis. As shown in Fig. 5, the prediction accuracy achieved by GAT for extracting protein structural features is 0.841, while that of APPNP stands at 0.826, and for GATV2 it is 0.844. Our TPepPro method yields the highest prediction accuracy of 0.855, highlighting its efficacy in extracting protein structural features.

### 3.4 Optimizing architecture of structural featuring neural network

**Figure 6.**
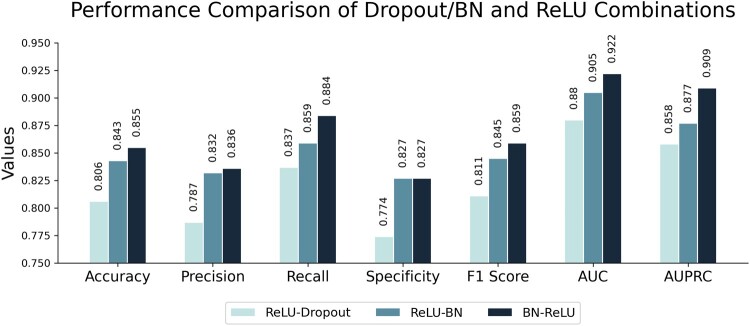
The combination of the Dropout/BN function and the ReLU function is used to evaluate the model performance sequentially.

In this study, the BN function and ReLU were used to reduce the overfitting of the model. Therefore, in this section, comparative experiments were conducted to compare with other functions: ReLU-Dropout, ReLU-BN, and BN_ReLU. The results, as depicted in Fig. 6, show that the BN-ReLU combination achieved the best performance.

### 3.5 Comparison with state-of-the-art methods

The baseline method was outperformed by the TPepPro model in binary interaction prediction. TPepPro focuses primarily on the binary classification of whether query peptide and the receptor protein interact with each other or not. In this study, the classification performance of TPepPro was compared with other state-of-the-art baseline methods, including the TAGPPI model based on deep learning using local sequence features and structural features of proteins for predicting PPIs (Song *et al.* 2022) the PIPR model based on deep learning for PPI prediction, and a Siamese Convolutional Neural Network (SCNN) model that excludes GRUs from PIPR (Chen *et al.* 2019). All prediction methods were evaluated on a benchmark dataset, with the four models assessed on the protein-peptide complex dataset (Lei *et al.* 2021). As indicated in Table 2, the TPepPro model achieved higher accuracy and AUC values for predicting PepPIs compared to the TAGPPI model (Song *et al.* 2022).

**Table 2.** Comparison of TPepPro model with three other models on protein-peptide complex datasets.

Model	Accuracy	Precision	Recall	Specificity	F1-score	AUC	AUPRC
PIPR	0.755	0.741	0.785	0.771	0.762	0.816	0.785
SCNN	0.729	0.722	0.745	0.737	0.734	0.790	0.753
TAGPPI	0.774	0.751	0.824	0.724	0.785	0.844	0.819
TPepPro (ours)	**0.855**	**0.836**	**0.884**	**0.827**	**0.859**	**0.922**	**0.909**

Note: Bold font indicates the best result in the column.

### 3.6 ROC curve and PR curve of various datasets with different methods

**Figure 7.**
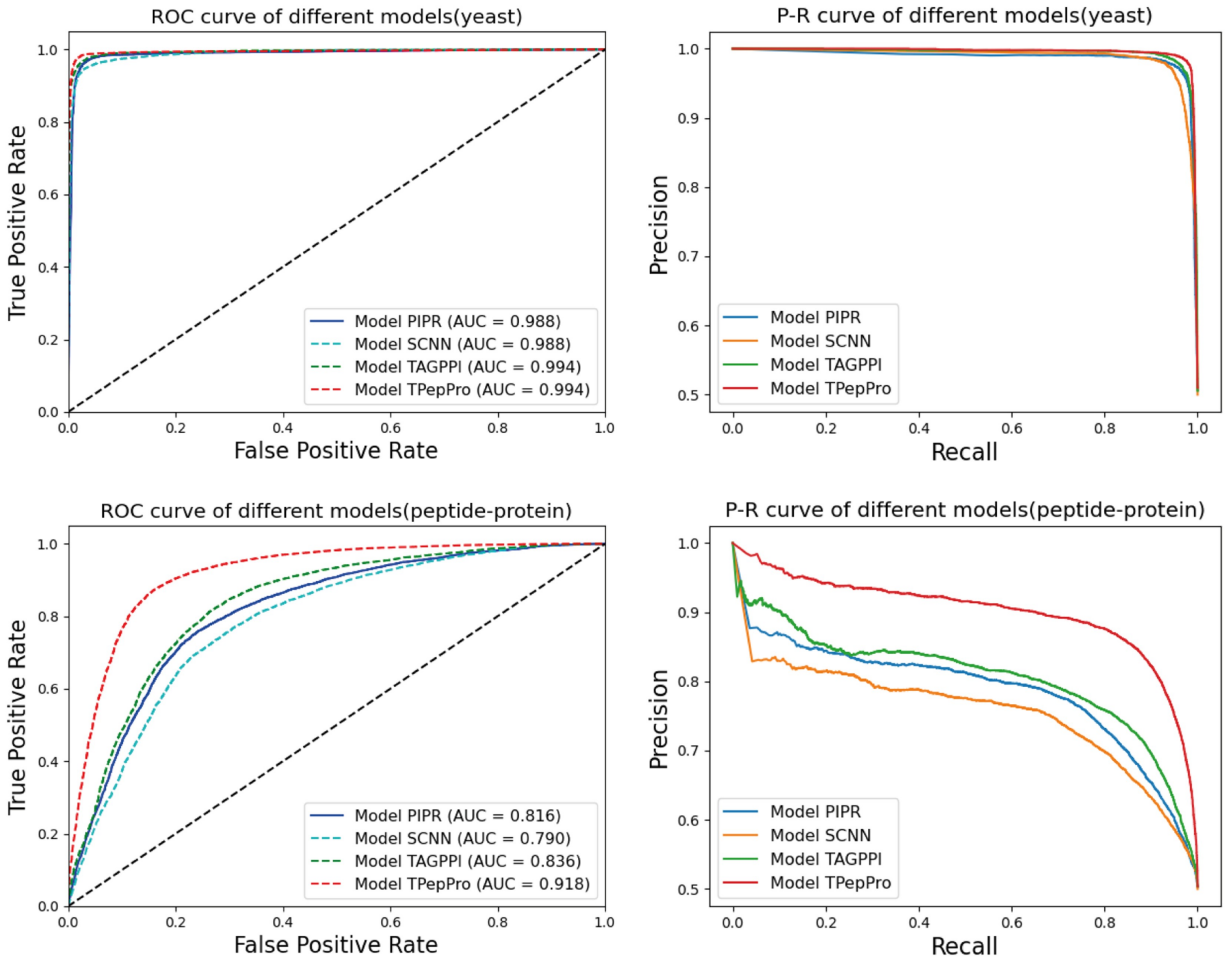
ROC curves and P-R curves of datasets. Curves of yeast dataset represent datasets that already have good performance in other methods. Curves protein-peptide complex dataset represents the pattern of datasets that do not have satisfying performance in other methods, yet greatly improved by TPepPro.

We compared the performance of four models, PIPR, SCNN, TAGPPI, and TPepPro. In multiple datasets, encompassing protein-peptide complex, human, yeast, HIV-human protein, and SARS-CoV-2-human protein datasets. Five-fold cross-validation was executed on each dataset, and the predictions from the five test sets generated for each model were aggregated. Subsequently, ROC (Receiver Operating Characteristic) curves and P-R (Precision-Recall) curves were generated for each dataset. To better illustrate the improvement in the performance of TPepPro by comparing it with other methods, the five datasets were divided into two groups to represent two different situations (Fig. 7). The first situation is exemplified by the yeast dataset, where existing models already perform well, with a mean AUC of 0.990 among the three existing models. TPepPro still ranks highly with an AUC of 0.994. Similar results were observed in the human Protein dataset and the HIV-human dataset (Supplementary Fig. S1). The other situation involves datasets where existing models perform unsatisfactorily in predicting PepPIs yet show great improvement with TPepPro. These datasets include the Propedia protein-peptide complex and the SARS-CoV-2-human protein datasets (Supplementary Fig. S2). From the ROC and PR curves, we found superior overall performance of TPepPro model exhibits in prediction across all five datasets as compared to the other three models.

### 3.7 Evaluation TPepPro performance on multiple datasets

To validate the applicability of TPepPro in other datasets, we conducted additional evaluations using human, yeast, and virus-human protein datasets. Such evaluations help us gain a more comprehensive understanding of TPepPro performance and its applicability cross different organisms. For evaluation of different models on these diverse datasets, we can more accurately evaluate their capabilities and accuracy, thereby facilitating its better application in real-world scenarios.

Tables 3–6 correspond to the experimental testing of TPepPro and other methods using human proteins, yeast, HIV-human proteins, and SARS-CoV-2-human protein datasets, respectively. From the experimental results, we conclude that TPepPro not only performs better on the protein-peptide complex dataset but also exhibits significantly better prediction performance compared to other models. This pattern is repeated on all five datasets. This indicates that TPepPro can achieve excellent prediction from multiple datasets, and further displays its reliability.

**Table 3.** Comparison of TPepPro with three other models on human datasets.

Model	Accuracy	Precision	Recall	Specificity	F1-score	AUC	AUPRC
PIPR	0.849	0.860	0.833	0.839	0.847	0.941	0.933
SCNN	0.937	0.927	0.948	**0.947**	0.937	0.983	0.981
TAGPPI	0.937	0.923	**0.953**	0.920	0.938	0.980	0.978
TPepPro (ours)	**0.947**	**0.943**	**0.953**	0.941	**0.948**	**0.988**	**0.989**

Note: Bold font indicates the best result in the column.

**Table 4:** Comparison of TPepPro with three other models on yeast datasets.

Model	Accuracy	Precision	Recall	Specificity	F1-score	AUC	AUPRC
PIPR	0.966	0.960	0.973	0.973	0.966	0.988	0.986
SCNN	0.958	0.963	0.952	0.952	0.957	0.988	0.986
TAGPPI	0.971	0.973	0.968	0.973	0.970	0.993	0.994
TPepPro (ours)	**0.979**	**0.981**	**0.978**	**0.981**	**0.979**	**0.994**	**0.994**

Note: Bold font indicates the best result in the column.

**Table 5.** Comparison of TPepPro with three other models on HIV-human protein datasets.

Model	Accuracy	Precision	Recall	Specificity	F1-score	AUC	AUPRC
PIPR	0.867	0.863	0.872	0.871	0.867	0.906	0.899
SCNN	0.875	0.886	0.861	0.865	0.873	0.933	0.935
TAGPPI	0.877	0.878	0.877	0.877	0.877	0.944	0.945
TPepPro (ours)	**0.891**	**0.886**	**0.897**	**0.884**	**0.891**	**0.956**	**0.954**

Note: Bold font indicates the best result in the column.

**Table 6.** Comparison of TPepPro with three other models on SARS-CoV-2-human protein datasets.

Model	Accuracy	Precision	Recall	Specificity	F1-score	AUC	AUPRC
PIPR	0.663	0.662	0.667	0.665	0.664	0.679	0.649
SCNN	0.671	0.666	0.683	0.675	0.674	0.717	0.685
TAGPPI	0.703	0.692	0.738	0.667	0.713	0.776	0.742
TPepPro (ours)	**0.747**	**0.733**	**0.807**	**0.687**	**0.759**	**0.833**	**0.821**

Note: Bold font indicates the best result in the column.

In addition, the AUCs obtained from the five datasets differ from those in the ROC curves. This is because the TAGPPI and TPepPro models calculate the AUCs separately for each fold using the predicted fold, and then sums these AUCs. Their average is treated as the final AUC value. However, the AUCs calculated for ROC curves are obtained by combining the predictions of the five testing sets before computation. In contrast, the AUCs in the tables from the PIPR and SCNN models are the same as those obtained in the ROC curves because their AUCs also use the same approaches, i.e. combined predictions of the five sets before evaluation.

Furthermore, four times of 5-fold cross-validations have been performed to increase the reliability of the tests. The results from each round of the validation are remarkably similar cross each fold. Moreover, the mean value of each evaluation parameter is very close to each other as well (Supplementary Table S5). This data indicates the stability and reliability of the TPepPro model.

### 3.8 Interpretability analysis

**Figure 8.**
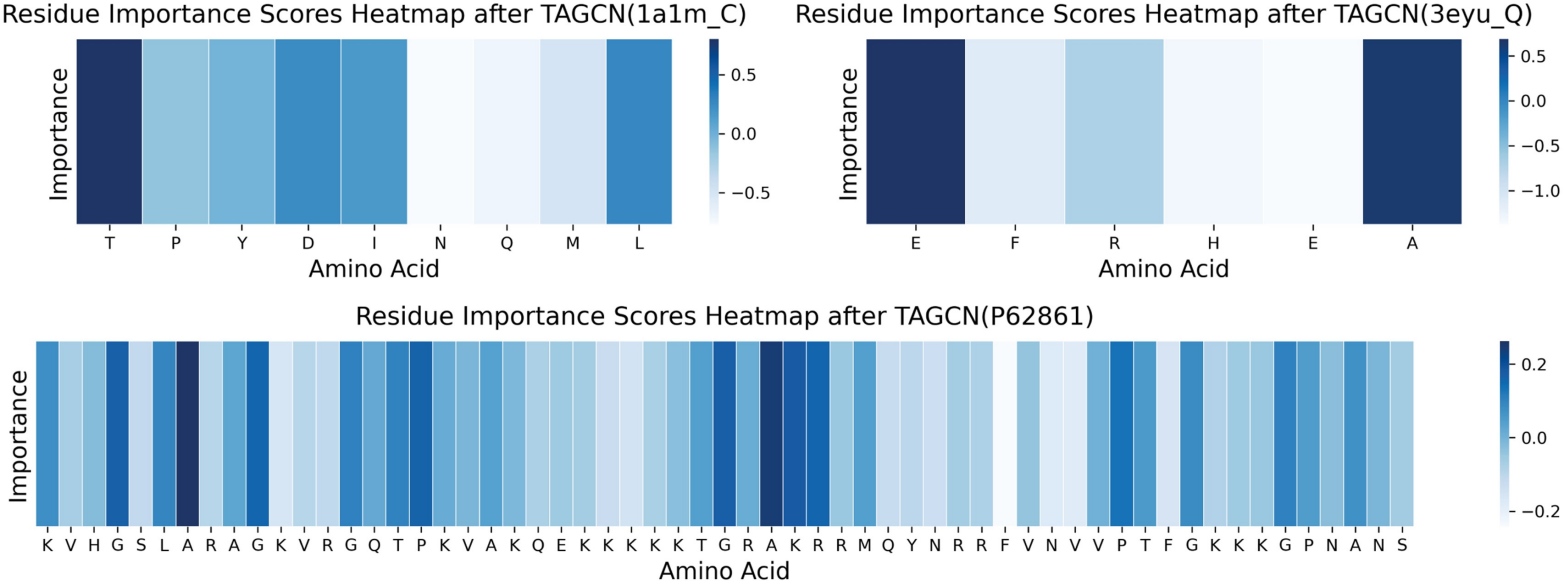
Heatmap of residue importance scores for global features of protein structures extracted by TAGCN.

The protein structures extracted using TAGCN were analyzed for interpretability of key global features. Taking peptides 3eyu_Q, 1a1m_C, and protein P62861 as an example. The node importance scores of each protein or peptide are shown in Fig. 8. The first amino acid E and the last amino acid A in 3eyu_Q, the first amino acid T in 1a1m_C and the seventh amino acid A in Protein P62861 show its importance in their respective sequences. This result represents a high potential for these amino acid sites that are likely to become peptide-protein binding sites.

**Figure 9.**
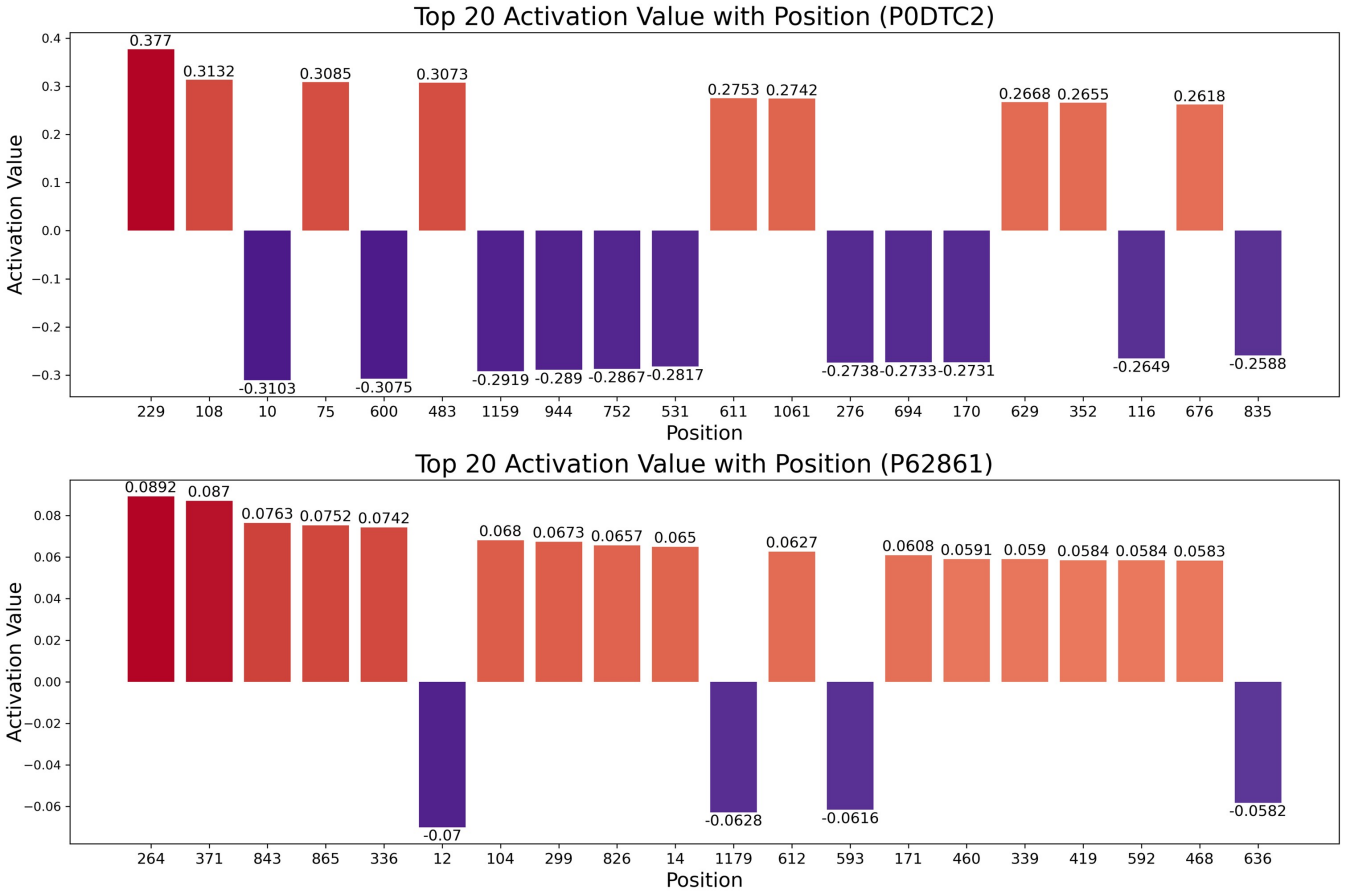
Activation map of local features of protein sequences extracted by TextCNN of protein P0DTC2 and P62861.

The TextCNN module extracts protein local sequence features from high-dimensional protein global features that have been encoded by the attention based ProtTrans model. Therefore, TextCNN lacks a direct attention mechanism to retrieve specific positions of the input sequences for interpretability analysis of protein local sequence features extracted by TextCNN. Therefore, the importance scores corresponding to specific amino acids cannot be derived. Here, we visualize the input vectors after passing through the first convolutional layer instead. This is because the data representations in post-first convolution establish a more intuitive connection with the original input data. Using the P0DTC2 and P62861 proteins as examples, the top 20 absolute values of activation values correspond to the activation maps shown in Fig. 9. The darker the color and the further left the position in the ranking, the higher the absolute value of the Activation Value. The red bars indicate positions with high Activation Values, suggesting a greater potential for these sites to serve as binding sites for PepPIs. Conversely, the more purple the color and the further left the ranking of the bars, the smaller the Activation Value, indicating that these positions are relatively conserved and less likely to become interaction binding sites.

### 3.9 TPepPro can find valid new interactions

From the predictions, we found 1662 pairs of new PepPIs that are not present in the input dataset before (Supplementary Table S2). The output of the TPepPro model contains two confidence values, which respectively refer the extent to which the model supports the presence (positive confidence value) or absence (negative confidence value) of interactions between sample pairs. Sixty-nine of them were found to have a positive confidence value greater than or equal to 0.99999, and more than 43% belong to humans. *Homo sapiens* was chosen as the organism for case studies for both peptide and protein, as we aim to find biologically meaningful evidence. However, the pairs involving to different species, including interactions between humans and various viruses or bacteria, are also present in the predictions and are worthy of investigation.

To validate the efficacy of the model and assess the practical biological relevance of the predicted outcomes, extensive research was conducted on the predicted results. Utilizing our custom-built package named TPepPro-filter for screening, 11 pairs of newly discovered high-confidence PepPIs were extracted. Their original label in the database was 0 (no interaction) and the predicted label was 1 (interaction exits). They all belong to *H.sapiens* with a confidence level greater than or equal to 0.99999 (Supplementary Table S3). Docking for each pair was carried out by ClusPro 2.0 to predict the interaction structure (Kozakov *et al.* 2017). All those 11 pairs were predicted to exhibit interaction. Docking results can be found in Supplementary Material S4 and Supplementary Fig. S3.

The dataset used in this study is Propedia v2.3, which collected published PepPIs from the Protein Data Bank on 15 November 2022. Therefore, the so-called new interactions here include those not recorded in Propedia, as well as truly novel interactions. Two cases of the former were found through extensive database searching and literature research. The two pairs of interactions were validated with experimental evidence (Table 7). At the same time, both pairs are ranked the highest positive confidence value among all human samples.

**Table 7.** Predicted new interactions which have experimental evidence.

Receptor	Peptide	Class_label	Confidence_positive	Predicted_label	Receptor Gene	Peptide Gene
6v13_B	4ov5_L	0	0.999999404	1	HLA-DRB1	HLA-A
3r85_B	7m5c_B	0	0.999998212	1	BCL2L	BAK

The first pair of interaction 6v13_B (PDB ID of HLA-DRB1) and 4ov5_L (HLA-A) was proved by Affinity Capture-Mass Spectrometry experiment, which originated from a pre-published dataset from Steve Huttlin *et al.* at Harvard Medical School (Huttlin *et al.* 2015). The second pair of experimentally confirmed interactions was between 3r85_B (BCL2L1) and 7m5c_B (BAK), which was more extensively reported. According to the database BioGRID 4.4 (Oughtred *et al.* 2021), there were 6 high-throughput and 10 low-throughput experimental evidences of the interaction between BCL2L1 and BAK (Griffiths *et al.* 1999, Degterev *et al.* 2001, Zhang *et al.* 2002, Whitfield *et al.* 2003, Rual *et al.* 2005, Willis *et al.* 2005, Venkatesan *et al.* 2009, Rudner *et al.* 2011, Trepte *et al.* 2015, Trepte *et al.* 2018, He *et al.* 2020, Luck *et al.* 2020, Huttlin *et al.* 2021, Li *et al.* 2021). Detailed experiments include Affinity Capture-Luminescence, Affinity Capture-Mass Spectrometry, Affinity Capture Western Blotting, Fluorescence Resonance Energy Transfer, PepPIs, Reconstituted Complex Assay, and Two-Hybrid Assay. Moreover, evidence of BCL2L2-BAK interaction was found as well, including two high-throughput experimental test using Two-Hybrid Assay (Holmgreen *et al.* 1999, Kim *et al.* 2014) and two high-throughput experiment using Affinity Capture-Western (Rolland *et al.* 2014, Luck *et al.* 2020).

The two validated pairs provide evidence to support the effectiveness of our predictive model. The remaining nine pairs have all passed the docking test, demonstrating a wide range/scope of potential interactions that have yet to be experimentally detected. Interestingly, four pairs in the results showed no interaction in the initial dataset (original label was 0), yet TPepPro predicted their existence with 100% confidence (Supplementary Table S3 Sheet2). It is worth noting that the first pair involves human protein and peptide from *Saccharomyces cerevisiae*, and the fourth pair involves T4 bacteriophage and human. Therefore, the two pairs of novel interactions predicted by TPepPro hold the potential for further biological validation.

## 4 Discussion and conclusion

A Transformer-based module was utilized in TPepPro for data preprocessing. Convolutional structures were employed to simultaneously extract features from amino acid sequences and contact maps describing the spatial structure of proteins. Additionally, an overfitting prevention method, Batch Normalization, was applied in the prediction part of the model for PepPIs prediction. It was proved that the performance could be improved by replacing the Dropout function with BN. The model architecture in TPepPro improves the accuracy by 8.1% in predicting PepPIs, as compared with the second-best model TAGPPI.

It is noticed that although machine learning techniques possess powerful computational capabilities, the authenticity of *in silico* prediction has been under considerable debate. Identification of potential PepPIs is inherently complex and challenging. In our study, TPepPro model displays its effectiveness in discovery of novel PepPIs that have been experimentally validated. A total of 17 experimental evidence were reported to support the accuracy of TPepPro system. Therefore, TPepPro not only outperforms previously published models in terms of accuracy, AUC, and other statistical metrices, but it also demonstrates experimental feasibility.

The interpretability analysis of the key global features extracted by TAGCN reveals amino acids with strong feature importance, such as first amino acid E in 3eyu_Q. This finding is significant for predicting peptide-protein binding sites. Similar feature importance is also observed in the local sequence features of TextCNN. Unfortunately, the dimensionality reduction performed by the convolutional layers hampers the mapping back to the original amino acid sequences. However, mapping the activation values back to the original amino acids would provide substantial biological insights. Ultimately, the models we explore will relate back to biological questions regarding active and contact sites between peptides and proteins. Identifying amino acids with the highest potential to become binding sites, along with relatively conserved amino acid positions, will be meaningful for studying protein-protein, protein-peptide, and peptide-peptide interactions. Therefore, in the future, we will develop methods for extracting local sequence features of proteins that can be traced back to amino acids. We aim to conduct a detailed interpretability analysis of both local features of protein sequences and global features of protein structures.

Furthermore, the PepPI model can be applied in drug research. Researchers have implemented the pre-trained TPepPro model on drug-target datasets. The resulting drug-target interaction prediction model will possess the capability to uncover novel interactions, to predict binding affinities, and to identify potential drug candidates with high specificity. By leveraging these capabilities, we can enhance the efficiency of drug discovery processes and pave the way for targeted therapies.

## Supplementary Material

btae708_Supplementary_Data

## Data Availability

Source code of model TPepPro is available at: https://github.com/wanglabhku/TPepPro. A package to manipulate TPepPro output files is available at https://github.com/wanglabhku/TPepPro filter. The TPepPro-filter package allows users to filter results based on specific confidence thresholds and desired combinations of species. The datasets used in this research were sourced from a peptide-protein interactions database located at: http://bioinfo.dcc.ufmg.br/propedia2/index.php/download.

## References

[btae708-B1] Ballester PJ , Mitchell JBO. A machine learning approach to predicting protein-ligand binding affinity with applications to molecular docking. Bioinformatics 2010;26:1169–75. 20236947 10.1093/bioinformatics/btq112 PMC3524828

[btae708-B2] Brody S, Alon U, Yahav E. How attentive are graph attention networks? arXiv, 10.48550/arXiv.2105.14491, 2022, preprint: not peer reviewed.

[btae708-B3] Chen M , Ju CJ-T, Zhou G et al Multifaceted protein-protein interaction prediction based on Siamese residual RCNN. Bioinformatics 2019;35:i305–14. 31510705 10.1093/bioinformatics/btz328 PMC6681469

[btae708-B4] Cole JC , Murray CW, Nissink JWM et al Comparing protein-ligand docking programs is difficult. Proteins 2005;60:325–32. 15937897 10.1002/prot.20497

[btae708-B5] Cunningham JM , Koytiger G, Sorger PK et al Biophysical prediction of protein–peptide interactions and signaling networks using machine learning. Nat Methods 2020;17:175–83. 31907444 10.1038/s41592-019-0687-1 PMC7004877

[btae708-B6] Degterev A , Lugovskoy A, Cardone M et al Identification of small-molecule inhibitors of interaction between the BH3 domain and Bcl-xL. Nat Cell Biol 2001;3:173–82. 11175750 10.1038/35055085

[btae708-B7] Du J, Zhang S, Wu G et al Topology adaptive graph convolutional networks. arXiv, 10.48550/arXiv.1710.10370, 2018, preprint: not peer reviewed.

[btae708-B8] Elnaggar A , Heinzinger M, Dallago C et al ProtTrans: toward understanding the language of life through self-supervised learning. IEEE Trans Pattern Anal Mach Intell 2022;44:7112–27. 34232869 10.1109/TPAMI.2021.3095381

[btae708-B9] Garbin C , Zhu X, Marques O et al Dropout vs. batch normalization: an empirical study of their impact to deep learning. Multimed Tools Appl 2020;79:12777–815.

[btae708-B10] Gasteiger J, Bojchevski A, Günnemann S. Predict then propagate: graph neural networks meet personalized PageRank. arXiv, 10.48550/arXiv.1810.05997, 2022, preprint: not peer reviewed.

[btae708-B11] Griffiths GJ , Dubrez L, Morgan CP et al Cell damage-induced conformational changes of the pro-apoptotic protein bank in vivo precede the onset of apoptosis. J Cell Biol 1999;144:903–14. 10085290 10.1083/jcb.144.5.903 PMC2148192

[btae708-B12] Hamelryck T , Manderick B. PDB file parser and structure class implemented in python. Bioinformatics 2003;19:2308–10. 14630660 10.1093/bioinformatics/btg299

[btae708-B13] He Y , Li W, Lv D et al Inhibition of USP7 activity selectively eliminates senescent cells in part via restoration of p53 activity. Aging Cell 2020;19:e13117. 32064756 10.1111/acel.13117 PMC7059172

[btae708-B14] Heinzinger M , Elnaggar A, Wang Y et al Modeling aspects of the language of life through transfer-learning protein sequences. BMC Bioinformatics 2019;20:723. 31847804 10.1186/s12859-019-3220-8 PMC6918593

[btae708-B15] Holmgreen SP , Huang DC, Adams JM et al Survival activity of Bcl-2 homologs Bcl-w and A1 only partially correlates with their ability to bind pro-apoptotic family members. Cell Death Differ 1999;6:525–32. 10381646 10.1038/sj.cdd.4400519

[btae708-B16] Huttlin EL , Bruckner RJ, Navarrete-Perea J et al Dual proteome-scale networks reveal cell-specific remodeling of the human interactome. Cell 2021;184:3022–40.e28. 33961781 10.1016/j.cell.2021.04.011 PMC8165030

[btae708-B17] Huttlin EL , Ting L, Bruckner RJ et al The BioPlex network: a systematic exploration of the human interactome. Cell 2015;162:425–40. 26186194 10.1016/j.cell.2015.06.043 PMC4617211

[btae708-B18] Ioffe S , Szegedy C. Batch normalization: accelerating deep network training by reducing internal covariate shift. arXiv, 10.48550/arXiv.1502.03167, 2015, preprint: not peer reviewed..

[btae708-B19] Johansson-Åkhe I , Mirabello C, Wallner B et al InterPep2: global peptide-protein docking using interaction surface templates. Bioinformatics 2020;36:2458–65. 31917413 10.1093/bioinformatics/btaa005 PMC7178396

[btae708-B20] Keeble AH , Turkki P, Stokes S et al Approaching infinite affinity through engineering of peptide-protein interaction. Proc Natl Acad Sci USA 2019;116:26523–33. 31822621 10.1073/pnas.1909653116 PMC6936558

[btae708-B21] Kim EM , Park JK, Hwang S-G et al Nuclear and cytoplasmic p53 suppress cell invasion by inhibiting respiratory complex-I activity via Bcl-2 family proteins. Oncotarget 2014;5:8452–65. 25115399 10.18632/oncotarget.2320 PMC4226696

[btae708-B22] Kim Y. Convolutional neural networks for sentence classification. In: *Proceedings of the 2014 Conference on Empirical Methods in Natural Language Processing (EMNLP)*, 2014, 1746–51. https://aclanthology.org/D14-1181 10.18653/v1/d16-1076 PMC5300751 28191551

[btae708-B23] Kozakov D , Hall DR, Xia B et al The ClusPro web server for protein-protein docking. Nat Protoc 2017;12:255–78. 28079879 10.1038/nprot.2016.169 PMC5540229

[btae708-B24] Lee AC-L , Harris JL, Khanna KK et al A comprehensive review on current advances in peptide drug development and design. Int J Mol Sci 2019;20:2383. 31091705 10.3390/ijms20102383 PMC6566176

[btae708-B25] Lei Y , Li S, Liu Z et al A deep-learning framework for multi-level peptide–protein interaction prediction. Nat Commun 2021;12:5465. 34526500 10.1038/s41467-021-25772-4 PMC8443569

[btae708-B26] Li W , Ma Y, He L et al Protease-activated receptor 2 stabilizes Bcl-xL and regulates EGFR-targeted therapy response in colorectal cancer. Cancer Lett 2021;517:14–23. 34098062 10.1016/j.canlet.2021.05.040

[btae708-B27] Li Z , Huang R, Xia M et al Fingerprinting interactions between proteins and ligands for facilitating machine learning in drug discovery. Biomolecules 2024;14:72. 38254672 10.3390/biom14010072 PMC10813698

[btae708-B28] Liu S , Liu C, Deng L. Machine learning approaches for protein−protein interaction hot spot prediction: progress and comparative assessment. Molecules 2018;23:2535. 30287797 10.3390/molecules23102535 PMC6222875

[btae708-B29] Luck K , Kim D-K, Lambourne L et al A reference map of the human binary protein interactome. Nature 2020;580:402–8. 32296183 10.1038/s41586-020-2188-x PMC7169983

[btae708-B30] Martins P , Mariano D, Carvalho FC et al Propedia v2.3: a novel representation approach for the peptide-protein interaction database using graph-based structural signatures. Front Bioinform 2023;3:1103103. 36875148 10.3389/fbinf.2023.1103103 PMC9978205

[btae708-B31] Oughtred R , Rust J, Chang C et al The BioGRID database: a comprehensive biomedical resource of curated protein, genetic, and chemical interactions. Protein Sci 2021;30:187–200. 33070389 10.1002/pro.3978 PMC7737760

[btae708-B32] Ramírez D , Caballero J. Is it reliable to use common molecular docking methods for comparing the binding affinities of enantiomer pairs for their protein target? Int J Mol Sci 2016;17:525. 27104528 10.3390/ijms17040525 PMC4848981

[btae708-B33] Rolland T , Taşan M, Charloteaux B et al A proteome-scale map of the human interactome network. Cell 2014;159:1212–26. 25416956 10.1016/j.cell.2014.10.050 PMC4266588

[btae708-B34] Rual J-F , Venkatesan K, Hao T et al Towards a proteome-scale map of the human protein-protein interaction network. Nature 2005;437:1173–8. 16189514 10.1038/nature04209

[btae708-B35] Rudner J , Elsaesser SJ, Jendrossek V et al Anti-apoptotic Bcl-2 fails to form efficient complexes with pro-apoptotic Bak to protect from Celecoxib-induced apoptosis. Biochem Pharmacol 2011;81:32–42. 20836993 10.1016/j.bcp.2010.09.002

[btae708-B36] Salwinski L , Miller CS, Smith AJ et al The database of interacting proteins: 2004 update. Nucleic Acids Res 2004;32:D449–51. 14681454 10.1093/nar/gkh086 PMC308820

[btae708-B37] Sinha K , Ghosh N, Sil PC et al A review on the recent applications of deep learning in predictive drug toxicological studies. Chem Res Toxicol 2023;36:1174–205. 37561655 10.1021/acs.chemrestox.2c00375

[btae708-B38] Song B , Luo X, Luo X et al Learning spatial structures of proteins improves protein-protein interaction prediction. Brief Bioinform 2022;23:bbab558. 35018418 10.1093/bib/bbab558

[btae708-B39] Sunny S , Jayaraj PB. Protein-protein docking: past, present, and future. Protein J 2022;41:1–26. 34787783 10.1007/s10930-021-10031-8

[btae708-B40] Tang T , Zhang X, Liu Y et al Machine learning on protein-protein interaction prediction: models, challenges and trends. Brief Bioinform 2023;24:bbad076. 10.1093/bib/bbad076 36880207

[btae708-B41] Trepte P , Buntru A, Klockmeier K et al DULIP: a dual luminescence-based co-immunoprecipitation assay for interactome mapping in mammalian cells. J Mol Biol 2015;427:3375–88. 26264872 10.1016/j.jmb.2015.08.003

[btae708-B42] Trepte P, Kruse S, Kostova S et al LuTHy: a double-readout bioluminescence-based two-hybrid technology for quantitative mapping of protein-protein interactions in mammalian cells. Mol Syst Biol 2018;14:e8071. 10.15252/msb.20178071 PMC6039870 29997244

[btae708-B43] Veličković P, Cucurull G, Casanova A et al. Graph attention networks. arXiv, 10.48550/arXiv.1710.10903, 2018, preprint: not peer reviewed.

[btae708-B44] Venkatesan K , Rual J-F, Vazquez A et al An empirical framework for binary interactome mapping. Nat Methods 2009;6:83–90. 19060904 10.1038/nmeth.1280 PMC2872561

[btae708-B45] Wang T, Xiong J, Xu X et al SCNN: a general distribution based statistical convolutional neural network with application to video object detection. In: *Proceedings of the AAAI Conference on Artificial Intelligence, 33 (No. 1: AAAI-19, IAAI-19, EAAI-20)*, 2019, 5321–8.

[btae708-B46] Whitfield J , Harada K, Bardelle C et al High-throughput methods to detect dimerization of Bcl-2 family proteins. Anal Biochem 2003;322:170–8. 14596824 10.1016/j.ab.2003.07.014

[btae708-B47] Willis SN , Chen L, Dewson G et al Proapoptotic Bak is sequestered by Mcl-1 and Bcl-xL, but not Bcl-2, until displaced by BH3-only proteins. Genes Dev 2005;19:1294–305. 15901672 10.1101/gad.1304105 PMC1142553

[btae708-B48] Yang A , Jude KM, Lai B et al Deploying synthetic coevolution and machine learning to engineer protein-protein interactions. Science2023a;381:eadh1720. 37499032 10.1126/science.adh1720 PMC10403280

[btae708-B49] Yang C, Xu X, Xiang C. Current computational methods for protein-peptide complex structure prediction. Curr Med Chem2023b;31:4058–78. 10.2174/0109298673263447230920151524 37888817

[btae708-B50] Yang X , Yang S, Lian X et al Transfer learning via multi-scale convolutional neural layers for human-virus protein-protein interaction prediction. Bioinformatics 2021;37:4771–8. 34273146 10.1093/bioinformatics/btab533 PMC8406877

[btae708-B51] Yin S, Mi X , Shukla D. Leveraging machine learning models for peptide-protein interaction prediction. RSC Chem Biol 2024;5:401–17. https://pubs.rsc.org/en/content/articlelanding/2024/cb/d3cb00208j. 10.1039/d3cb00208j PMC11078210 38725911

[btae708-B52] Zhai H , Hou H, Luo J et al DGDTA: dynamic graph attention network for predicting drug–target binding affinity. BMC Bioinformatics 2023;24:367. 37777712 10.1186/s12859-023-05497-5 PMC10543834

[btae708-B53] Zhang H , Nimmer P, Rosenberg SH et al Development of a high-throughput fluorescence polarization assay for Bcl-xL. Anal Biochem 2002;307:70–5. 12137781 10.1016/s0003-2697(02)00028-3

[btae708-B54] Zhao N , Zhuo M, Tian K et al Protein–protein interaction and non-interaction predictions using gene sequence natural vector. Commun Biol 2022;5:652. 35780196 10.1038/s42003-022-03617-0 PMC9250521

