# Supplementary material for: TPepPro: a deep learning model for predicting peptide–protein interactions
Source: Bioinformatics. 2024 Nov 25;41(1):btae708. doi: 10.1093/bioinformatics/btae708 (PMC11681936; doi:10.1093/bioinformatics/btae708)
Supplement: btae708_Supplementary_Data [file btae708_supplementary_data.zip › Supplementary Table S1.docx]

Table S1. Five datasets for the TBTPpepI method and other methods

| Data type | Data meaning | Positive sample data source | Negative sample data source |
| --- | --- | --- | --- |
| Protein-peptide | Used to evaluate the performance of the model in predicting protein-peptide interactions. The dataset contains 19187 pairs of data, with 9594 pairs of interacting protein-peptide interactions and 9593 pairs of non-interacting protein-peptide pairs. | A database of protein-peptide interactions(URL:http://bioinfo.dcc.ufmg.br/propedia2/index.php/download） | Negative samples are constructed by shuffling positive sample pairs and selecting sample pairs that do not appear in the positive samples |
| Human | Used to evaluate the performance of the model in predicting human protein-protein interactions. The dataset contains 2320 pairs of data, with 1160 pairs of interacting human protein-protein interactions and 1160 pairs of non-interacting human protein-protein pairs. | The Database of Interacting Proteins(DIP:[https://dip.doe-mbi.ucla.edu/dip/](https://dip.doe-mbi.ucla.edu/dip/)) | Negatome Database 2.0 (http://mips.helmholtz-muenchen.de/proj/ppi/negatome) |
| Yeast | Used to evaluate the performance of the model in predicting yeast protein-protein interactions. The dataset contains 11188 pairs of data, with 5594 pairs of interacting yeast protein-protein interactions and 5594 pairs of non-interacting yeast protein-protein pairs. | The Database of Interacting Proteins(http://dip.doe-mbi.ucla.edu) | Negative interactions  were generated by randomly pairing the proteins without  evidence of interaction |
| HIV-human | Used to evaluate the performance of the model in predicting HIV-human protein interactions. The dataset contains 3080 pairs of data, with 1540 pairs of interacting HIV-human protein pairs and 1540 pairs of non-interacting HIV-human protein pairs. | Five public databases:HPIDB( URL：http://www.agbase.msstate.edu/hpi/main.html),  VirHostNet(URL: (http://virhostnet.prabi.fr) ,VirusMentha (URL:http://virusmentha.uniroma2.it/), PHISTO(URL:http://www.phisto.org) and PDB(URL:http://rcsb.org) | The negative samples were sampled using the "difference-based negative sampling" method |
| SASR-CoV-2-h  uman | Used to evaluate the performance of the model in predicting SARS-CoV-2-human protein interactions. The dataset contains 1100 pairs of data, with 550 pairs of interacting SARS-CoV-2-human protein pairs and 550 pairs of non-interacting SARS-CoV-2-human protein pairs. | Two high-throughput MS experiments | The negative samples were sampled using the "difference-based negative sampling" method |
