# Supplementary material for: TPepPro: a deep learning model for predicting peptide–protein interactions
Source: Bioinformatics. 2024 Nov 25;41(1):btae708. doi: 10.1093/bioinformatics/btae708 (PMC11681936; doi:10.1093/bioinformatics/btae708)

Supplementary Figure 1. ROC curves and P-R curves of datasets that already have good performance in other methods. Datasets including yeast, human protein dataset, and HIV-human dataset.


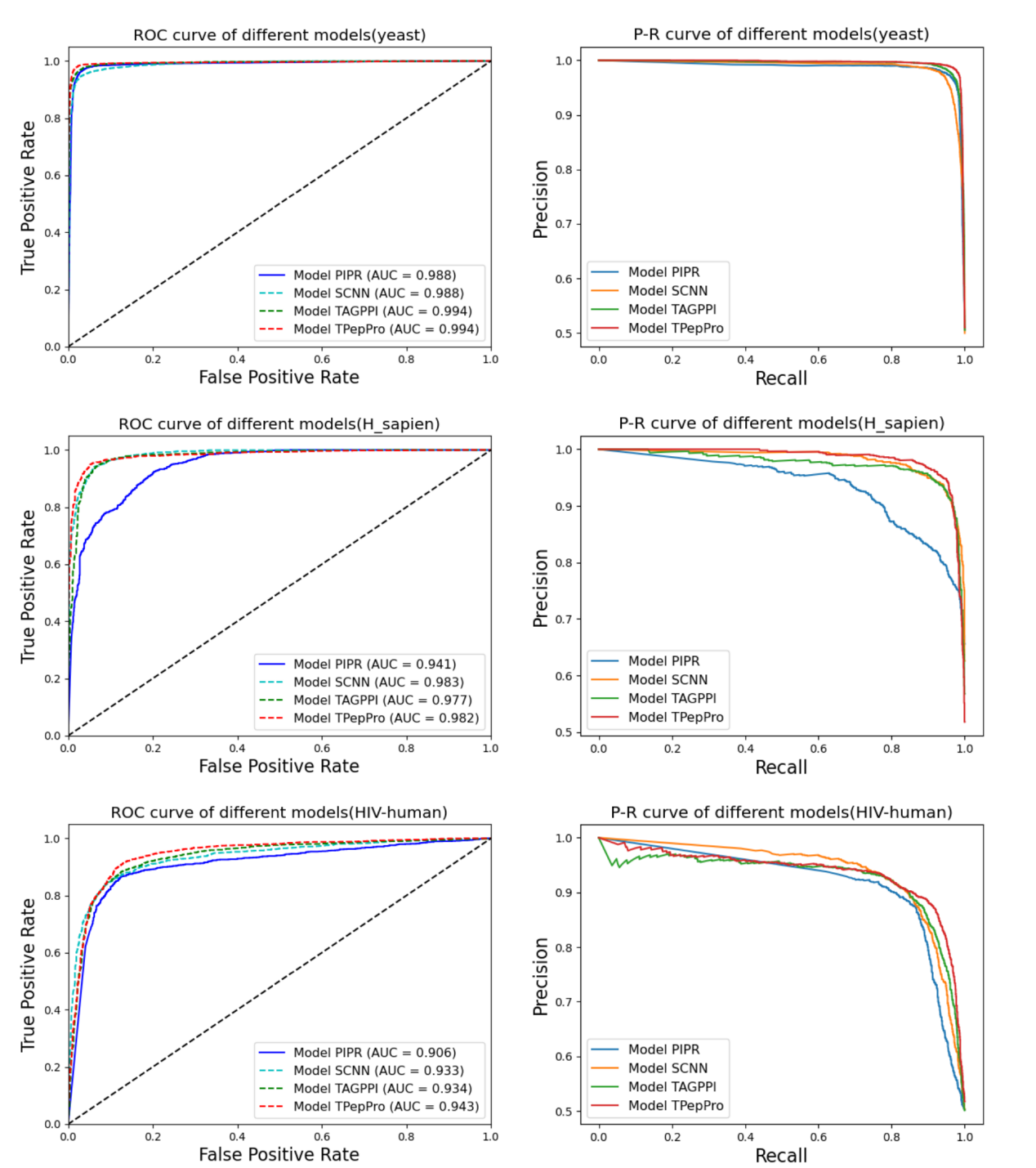


Supplementary Figure 2. ROC curves and P-R curves of datasets that do not have satisfying performance in other methods, yet greatly improved by TPepPro. Datasets including: Propedia protein-peptide complex dataset and SARS-CoV-2-human protein datasets.


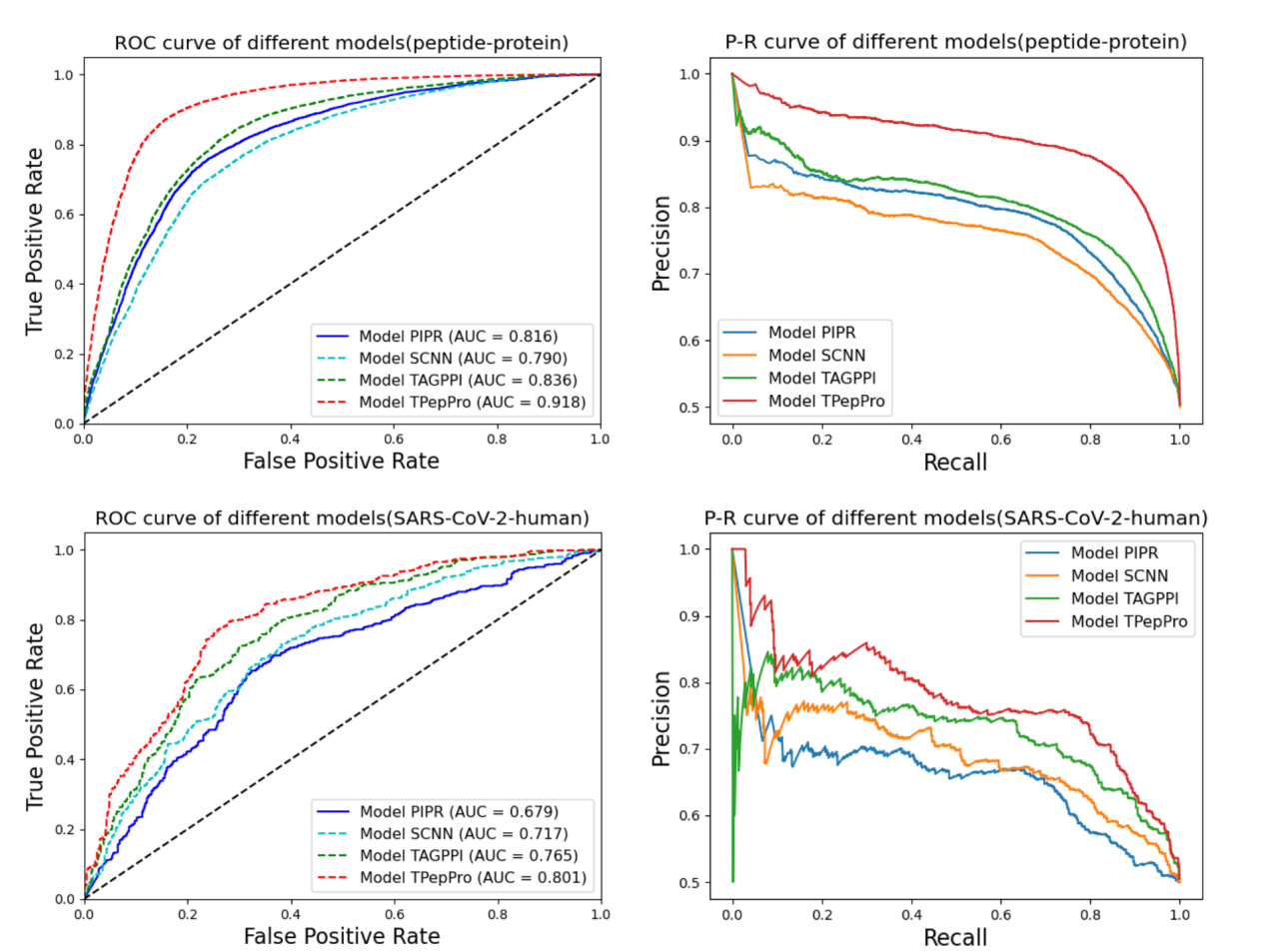


**Supplementary Figure 3.** Docking results of two experimentally proved new interactions. Chains as sticks indicate peptide. Chains in cartoons represent the receptor. Surface in red and blue shows a binding pocket with protein contact potential. (**A**) Docking structure between HLA-A (6v13_B) and HLA-DRB1 (4ov5_L). (**B**) Docking structure between BCL2L (3r85_B) and BAK (7m5c_B).


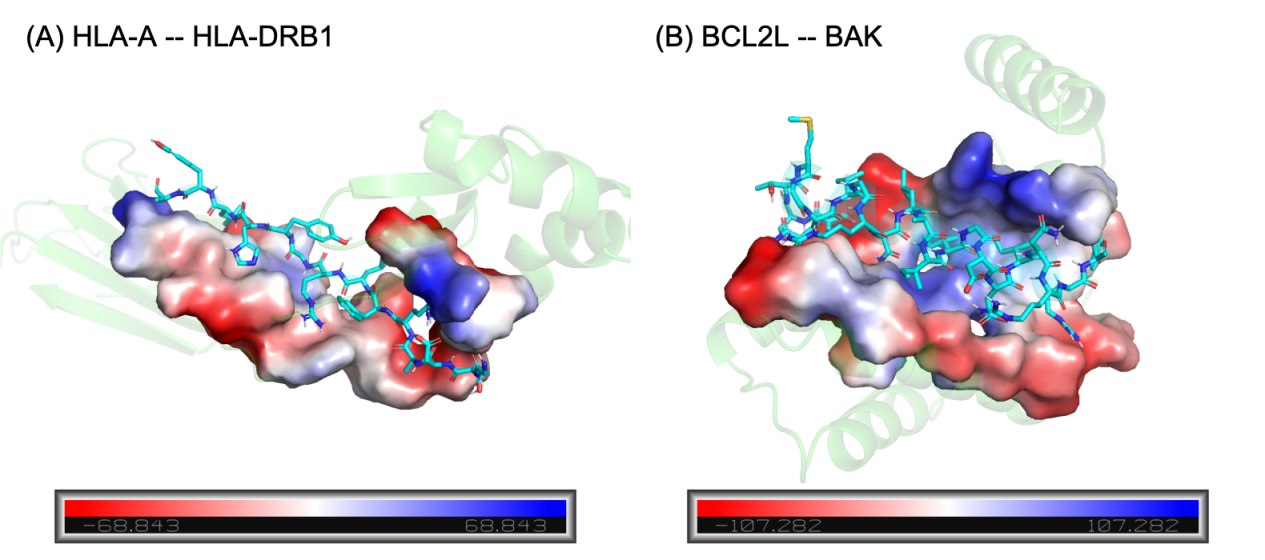

Supplement: btae708_Supplementary_Data [file btae708_supplementary_data.zip › Supplementary Figure S5.docx]
